# Regulation of Melanocortin-3 and -4 Receptors by Isoforms of Melanocortin-2 Receptor Accessory Protein 1 and 2

**DOI:** 10.3390/biom12020244

**Published:** 2022-02-02

**Authors:** Ren-Lei Ji, Ya-Xiong Tao

**Affiliations:** Department of Anatomy, Physiology and Pharmacology, College of Veterinary Medicine, Auburn University, Auburn, AL 36849, USA; rlj0027@auburn.edu

**Keywords:** melanocortin-2 receptor accessory protein 1, melanocortin-2 receptor accessory protein 2, melanocortin-3 receptor, melanocortin-4 receptor, pharmacology

## Abstract

The neural melanocortin receptors (MCRs), melanocortin-3 and -4 receptors (MC3R and MC4R), play essential non-redundant roles in the regulation of energy homeostasis. Interaction of neural MCRs and melanocortin-2 receptor accessory proteins (MRAPs, MRAP1 and MRAP2) is suggested to play pivotal roles in MC3R and MC4R signaling. In the present study, we identified two new human (h) *MRAP2* splice variants, *MRAP2b* (465 bp open reading frame) and *MRAP2c* (381 bp open reading frame). Human MRAP2s are different in C-termini. We investigated the effects of five isoforms of MRAPs, hMRAP1a, hMRAP1b, hMRAP2a, hMRAP2b, and hMRAP2c, on MC3R and MC4R pharmacology. At the hMC3R, hMRAP1a and hMRAP2c increased and hMRAP1b decreased the cell surface expression. hMRAP1a increased affinity to ACTH. Four MRAPs (hMRAP1a, hMRAP1b, hMRAP2a, and hMRAP2c) decreased the maximal responses in response to α-MSH and ACTH. For hMC4R, hMRAP1a, hMRAP2a, and hMRAP2c increased the cell surface expression of hMC4R. Human MRAP1b significantly increased affinity to ACTH while MRAP2a decreased affinity to ACTH. Human MRAP1a increased ACTH potency. MRAPs also affected hMC4R basal activities, with hMRAP1s increasing and hMRAP2s decreasing the basal activities. In summary, the newly identified splicing variants, hMRAP2b and hMRAP2c, could regulate MC3R and MC4R pharmacology. The two MRAP1s and three MRAP2s had differential effects on MC3R and MC4R trafficking, binding, and signaling. These findings led to a better understanding of the regulation of neural MCRs by MRAP1s and MRAP2s.

## 1. Introduction

Melanocortin receptors (MCRs), MC1R to MC5R, are members of rhodopsin-like Family A G-protein-coupled receptors (GPCRs) activated by melanocortin peptides including α-, β-, and γ-melanocyte-stimulating hormone (α-, β-, and γ-MSH) and adrenocorticotropic hormone (ACTH) [1,2]. MC3R and MC4R (neural MCRs) are primarily expressed in the central nervous system [3,4,5,6] and play pivotal roles in regulating energy homeostasis [7,8]. Mice lacking *Mc4r* display morbid obesity with decreased energy expenditure and increased food intake [9,10]. *Mc3r* knockout mice have moderate obesity phenotype with normal food intake and metabolism, increased fat mass, and decreased lean mass [11,12,13]. These results indicate distinct non-redundant mechanisms between MC3R and MC4R in regulating energy homeostasis. Furthermore, variants in *MC3R* and *MC4R* have been shown to be closely associated with monogenic human obesity [14,15,16,17,18]. In addition, MC4R is also involved in sexual function and reproduction [16,18]. MC3R is also expressed in the periphery and may have other potential physiological functions in regulating cardiovascular function [19,20], immune response [21,22,23,24,25], natriuresis [26], and timing of sexual maturation [27].

Melanocortin-2 receptor accessory protein 1 (MRAP1), first identified as low molecular weight protein from fat tissue [28], was the first MC2R accessory protein identified, as the specific molecular chaperone for MC2R in regulating receptor expression, ligand binding, and signaling [29,30,31,32]. *MRAP1* mutations account for ~20% of familial glucocorticoid deficiency cases [29,33]. There are two alternatively spliced isoforms of human (h) MRAP1, hMRAP1a and hMRAP1b, with similar effects on MC2R trafficking and signaling [29,30]. *MRAP1a* and *MRAP1b* are widely expressed, but their distribution patterns are distinct [29,34]. These results suggest that hMRAP1a and hMRAP1b might possess multiple functions beyond regulating MC2R (primarily expressed in the adrenal gland) [29]. Indeed, hMRAP1a has been shown to regulate all five hMCRs in distinct ways [35,36,37]. However, almost all of the investigations focus on MRAP1a and its regulation on MC3R/MC4R, and there are few studies on MRAP1b.

MRAP2 (a paralog of MRAP1) shares 40% homology with MRAP1 and has different functions from MRAP1s. MRAP2 with high expression in the brain is essential for the modulation of energy homeostasis. *Mrap2* knockout mice display early-onset severe obesity [38,39]. *MRAP2* mutations are associated with severe obesity in humans [38,40,41,42]. MRAP2 regulates MC3R or MC4R signaling in mammals and other species [35,38,43,44,45,46,47,48,49]. It has been reported that hMRAP2a either decreases [Nle4,D-Phe7]-α-MSH (NDP-MSH)-stimulated [35] or increases α-MSH-stimulated [50] cAMP generation of hMC3R and hMC4R. In addition, hMRAP2a increases ACTH potency of hMC4R [51]. In teleosts, there are two copies of *mrap2* (*mrap2a* and *mrap2b*) in zebrafish and topmouth culter with various modulatory roles of MCRs in these fishes [43,47,48].

Recently, we identified two new human *MRAP2* splice variants, *MRAP2b* and *MRAP2c*. Human MRAP2b and MRAP2c share the same amino acid sequences in N-termini and transmembrane domains (TMD) with hMRAP2a. However, whether MRAP2b and MRAP2c are involved in MC3R/MC4R regulation was unknown. Additionally, the regulation of MC3R/MC4R by MRAP1b is not clear. Hence, the potential effects of all five isoforms of hMRAPs, hMRAP1a, hMRAP1b, hMRAP2a, hMRAP2b, and hMRAP2c, on hMC3R and hMC4R pharmacology were systematically investigated in this study.

## 2. Materials and Methods

### 2.1. Ligands and Plasmids

NDP-MSH was obtained from Peptides International (now Vivitide, Louisville, KY, USA). *α*-MSH was purchased from Pi Proteomics (Huntsville, AL, USA). Human ACTH(1–24) was purchased from Phoenix Pharmaceuticals (Burlingame, CA, USA). [^125^I]-NDP-MSH and [^125^I]-cAMP were iodinated using chloramine T method [52,53]. The N-terminal myc-tagged human MC4R (hMC4R) subcloned into pcDNA3.1 vector was generated as previously described [54]. N-terminal myc-tagged hMC3R and N-terminal Flag-tagged hMRAP1a, hMRAP1b, hMRAP2a, hMRAP2b, and hMRAP2c were commercially synthesized by Synbio Technologies (Monmouth Junction, NJ, USA) to generate the plasmids used for transfection.

### 2.2. Cell Culture and Transfection

Human embryonic kidney (HEK) 293T cells were obtained from the American Type Culture Collection (Manassas, VA, USA) and were cultured at 37 °C in a 5% CO_2_-humidified atmosphere in Dulbecco’s Modified Eagle’s medium (DMEM) containing 10% newborn calf serum, 10 mM HEPES, 100 IU/mL of penicillin, 0.25 μg/mL of amphotericin B, 50 μg/mL of gentamicin, and 100 μg/mL streptomycin [54]. Cells were plated into gelatin-coated 24-well plates. Cells, when reaching 50–70% confluency, were co-transfected with 0.25 μg/μL hMC3R or hMC4R with or without MRAP1 or MRAP2 plasmids using the calcium phosphate precipitation method [55]. The total DNA was normalized using empty vector pcDNA3.1 in each well.

### 2.3. Flow Cytometry Assay

The influence of hMRAP1s or hMRAP2s on the total and cell surface expression of hMC3R and hMC4R was performed using flow cytometry (Accuri Cytometers, Ann Arbor, MI, USA) as described previously [56,57]. Cells (6-well plates) were transfected with hMC3R or hMC4R (N-terminal c-myc tag) and hMRAP1a, hMRAP1b, hMRAP2a, hMRAP2b or hMRAP2c plasmids at a ratio of 1:5. Fluorescence of cells transfected with empty vector (pcDNA3.1) was used for background staining. The expression of the hMC3R or hMC4R was calculated as the percentage of the cell transfected with hMC3R or hMC4R but without MRAPs set as 100% [56].

### 2.4. Radioligand Ligand Binding Assays

The binding assay was described previously [45,54]. To explore the regulation of hMRAP1s or hMRAP2s on the binding property of hMC3R or hMC4R, hMC3R or hMC4R (0.25 μg/μL) with hMRAP1 or hMRAP2 plasmids at a ratio of 1:5 was co-transfected into cells (24-well plate or 6-well plate). Two ligands, α-MSH (from 10^−12^ to 10^−5^ M) and ACTH(1–24) (from 10^−12^ to 10^−6^ M), were used in this study

### 2.5. Ligand-Stimulated cAMP Assays

cAMP signaling assay was performed by radioimmunoassay (RIA) as described previously [52,54,58]. Cells (24-well plate) were transfected with hMC3R or hMC4R (0.25 μg/μL) and hMRAP21s or hMRAP2s plasmids at a ratio of 1:5, and two ligands, α-MSH and ACTH(1–24), were used.

### 2.6. Statistical Analysis

All data were represented as mean ± S.E.M. The parameters and significance of differences were calculated by GraphPad Prism 8.3 software (GraphPad, San Diego, CA, USA). The significance of differences in ligand binding, cAMP signaling, and flow cytometry parameters were all determined by one-way ANOVA, with *p* < 0.05 set as significant.

## 3. Results

### 3.1. Nucleotide and Deduced Amino Acid Sequences of hMRAP2s

**Figure 1:**
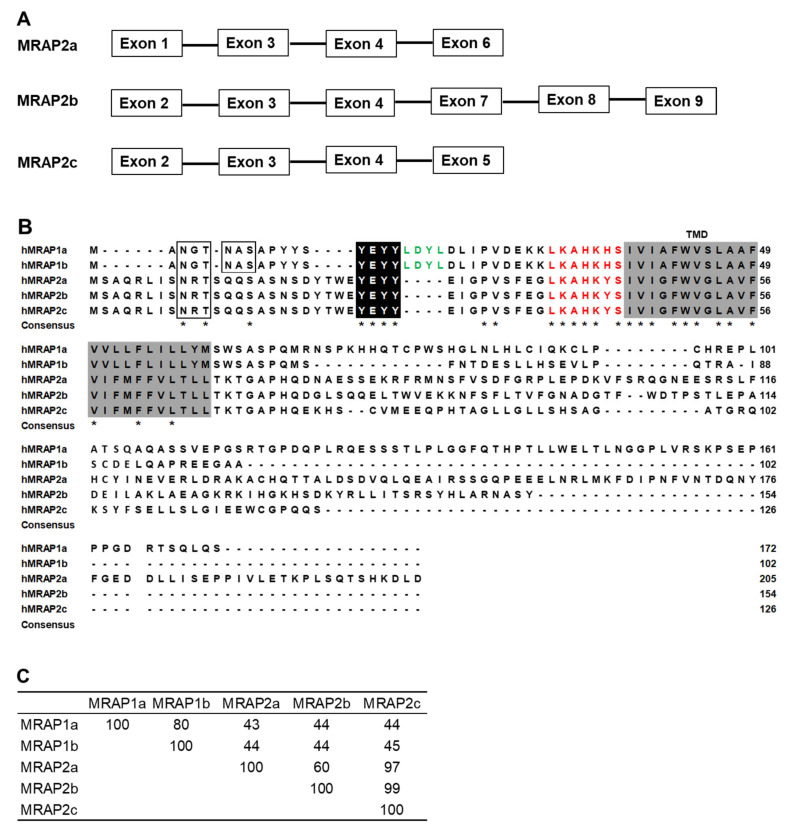
Schematic diagram of the human MRAP2 splice variants (**A**), comparison of amino acid sequences of human MRAP1s and MRAP2s (**B**), and amino acid sequence identities of MRAP1s and MRAP2s (**C**). Open boxes indicate potential N-linked glycosylation sites. Dark shadows show conserved motifs (YEYY) in both MRAP1 and MRAP2. The green color indicates the activation motif (LDYL). LKAHKHS (red color) in MRAP1s and LKAHKYS (red color) in MRAP2s are required for dual topology. Transmembrane domains are shown in shaded boxes. Asterisk (*) denotes the same amino acids.

Human *MRAP2* (NG_051944.1) is composed of 11 exons. Three *MRAP2* splice variants were identified: *MRAP2a* (XM_017010220.1) derived from 4 exons (1, 3, 4, and 6) that had 618 bp open reading frame (ORF), encoding a protein of 205 amino acids; *MRAP2b* (XM_017010221.2) derived from 6 exons (2, 3, 4, 7, 8, and 9) that had a 465 bp ORF, encoding a protein of 154 amino acids; and *MRAP2c* (XM_024446318.1) derived from 4 exons (2, 3, 4, and 5) that had a 381 bp ORF, encoding a protein of 126 amino acids (Figure 1A). Human MRAP2b and hMRAP2c had the same sequences in the N- termini, a putative LKAHKYS motif, and a single conserved transmembrane domain but different C-termini from hMRAP2a (Figure 1B). Two potential *N*-linked glycosylation sites (Asn^3^ and Asn^6^) in N-termini of hMRAP1a and hMRAP1b, and one potential *N*-linked glycosylation site (Asn^9^) in N-termini of hMRAP2a, hMRAP2b, and hMRAP2c (Figure 1B). In addition, a conserved motif (YEYY) was observed in all hMRAP1s and hMRAP2s (Figure 1B). LDYL motif was only present in hMRAP1s but not in hMRAP2s (Figure 1B). MRAP2a shared 60% amino acid identity to MRAP2b, and 97% identity to MRAP2c (Figure 1C). MRAP2b had 99% identity to MRAP2c (Figure 1C).

### 3.2. Regulation of hMC3R Pharmacology by hMRAP1s and hMRAP2s

**Figure 2:**
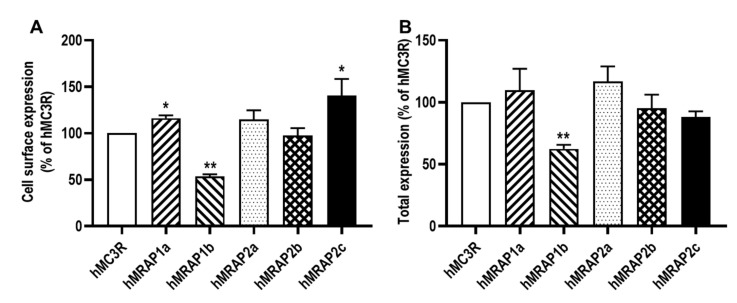
Regulation of hMC3R expression by hMRAP1s or hMRAP2s. Cell surface (**A**) and total (**B**) expression of hMC3R were measured by flow cytometry. HEK293T cells were co-transfected with hMC3R and hMRAP1s or hMRAP2s. Fluorescence in cells transfected with empty vector pcDNA3.1 was used for background staining. The results are calculated as % of 1:0 group. Each data point represented the mean ± SEM (n = 3). * indicates significant difference (* *p* < 0.05 and ** *p* < 0.01) (one-way ANOVA followed by Tukey test).

Flow cytometry was used to determine MRAP regulation of hMC3R expression (Figure 2). The results showed that hMRAP1a and hMRAP2c significantly increased the cell surface expression, and hMRAP1b decreased the cell surface expression of hMC3R (Figure 2A). Human MRAP2a and hMRAP2b had no effect on the cell surface expression of hMC3R (Figure 2A). Only hMRAP1b decreased the total expression of hMC3R, and the other four MRAPs did not affect the total expression of hMC3R (Figure 2B).

**Figure 3:**
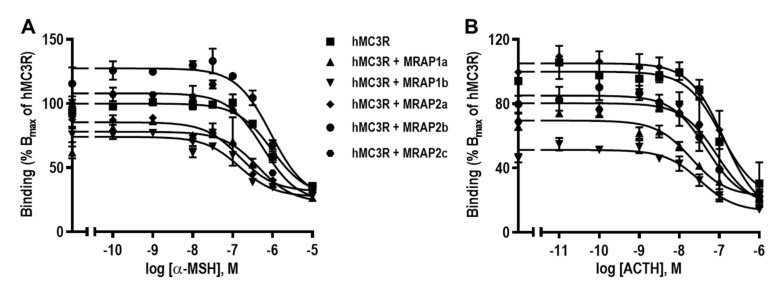
The ligand binding properties of hMC3R regulated by hMRAP1s or hMRAP2s to α-MSH (**A**) and ACTH (**B**). HEK293T cells were transiently transfected with hMC3R with or without hMRAP1a, hMRAP1b, hMRAP2a, hMRAP2b or hMRAP2c plasmids (1:5), and the binding properties were measured 48 h later by displacing the binding of ^125^I-NDP-MSH using different concentrations of unlabeled α-MSH and ACTH(1–24). Data are expressed as % of hMC3R binding ± range from duplicate measurements within one experiment. The curves are representative of at least three independent experiments.

Competitive ligand binding assays were performed to explore MRAP regulation of hMC3R binding properties. Different concentrations of unlabeled *α*-MSH or ACTH(1–24) were used to compete with a fixed amount of ^125^I-NDP-MSH. Results showed that only hMRAP1b significantly decreased the maximal binding value (B_max_), and hMRAP1a, hMRAP2a, hMRAP2b, and hMRAP2c had no significant effect on B_max_s of hMC3R (Figure 3 and Table 1). All MRAPs did not affect α-MSH affinities at hMC3R (Figure 3A and Table 1). Only hMRAP1a increased ACTH affinity of hMC3R, and the other MRAPs had no effect on affinities of hMC3R to ACTH (Figure 3B and Table 1).

**Figure 4:**
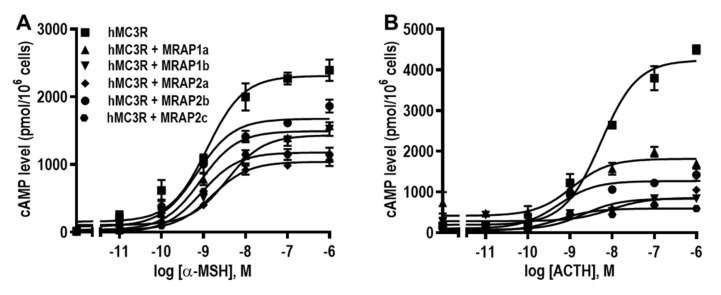
The signaling properties of hMC3R regulated by hMRAP1s or hMRAP2s in response to α-MSH (**A**) and ACTH (**B**). HEK293T cells were transiently transfected with hMC3R with or without hMRAP1a, hMRAP1b, hMRAP2a, hMRAP2b or hMRAP2c plasmids (1:5). Intracellular cAMP levels were measured by RIA after stimulation with different concentrations of α-MSH and ACTH(1–24). The curves are representative of at least three independent experiments. All experiments were performed at least three times independently.

The signaling properties of hMC3R modulated by MRAPs were determined using cAMP RIA. Results showed that all hMRAPs had no significant effect on potencies of hMC3R to α-MSH and ACTH (Figure 4A,B and Table 2). Four hMRAPs (hMRAP1a, hMRAP1b, hMRAP2a, and hMRAP2c) markedly decreased maximal responses (R_max_) in response to α-MSH and ACTH, and hMRAP2b deceased R_max_ to ACTH but not α-MSH (Figure 4A,B and Table 2). In addition, all MRAPs significantly decreased the basal activities of hMC3R (Table 2).

### 3.3. Regulation of hMC4R Pharmacology by hMRAP1s and hMRAP2s

**Figure 5:**
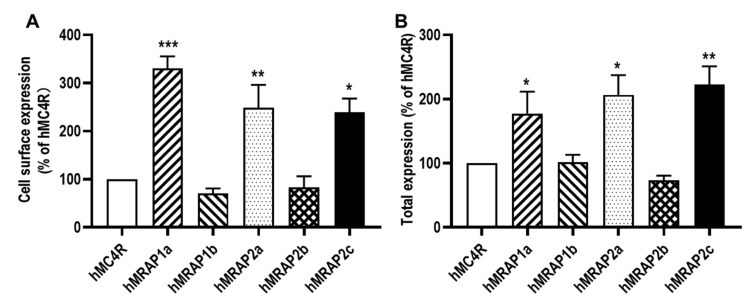
Regulation of hMC4R expression by hMRAP1s or hMRAP2s. Cell surface (**A**) and total (**B**) expression of hMC3R were measured by flow cytometry. HEK293T cells were co-transfected with hMC3R and hMRAP1s or hMRAP2s. Fluorescence in cells transfected with empty vector pcDNA3.1 was used for background staining. The results are calculated as % of 1:0 group. Each data point represented the mean ± SEM (n = 3). * indicates significant difference (* p < 0.05, ** p < 0.01, and *** p < 0.001) (one-way ANOVA followed by Tukey test).

Results of flow cytometry showed that hMRAP1a, hMRAP2a, and hMRAP2c significantly increased, while hMRAP1b and hMRAP2b had no significant effect on the cell surface and total expression of hMC4R (Figure 5).

**Figure 6:**
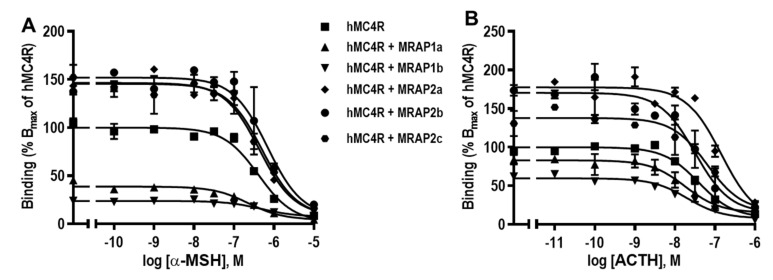
The ligand-binding properties of hMC4R regulated by hMRAP1s or hMRAP2s to α-MSH (**A**) and ACTH (**B**). HEK293T cells were transiently transfected with hMC4R with or without MRAP1a, MRAP1b, MRAP2a, MRAP2b or MRAP2c plasmids (1:5), and the binding properties were measured 48 h later by displacing the binding of ^125^I-NDP-MSH using different concentrations of unlabeled α-MSH and ACTH(1–24). Data are expressed as % of hMC4R binding ± range from duplicate measurements within one experiment. All experiments were performed at least three times independently.

Ligand binding assays indicated that at hMC4R, hMRAP1a and hMRAP1b significantly decreased B_max_s, while hMRAP2a increased B_max_s (Figure 6 and Table 3). No significant effect was observed for hMRAP1a, hMRAP1b, hMRAP2a, hMRAP2b, and hMRAP2c on α-MSH affinities at the hMC4R (Figure 6A and Table 3). Additionally, hMRAP1b increased affinity, whereas hMRAP2a decreased affinity of hMC4R to ACTH (Figure 6B and Table 4). MRAP1a, MRAP2b, and MRAP2c had no effects on affinities of hMC4R to ACTH (Figure 6B and Table 3).

**Figure 7:**
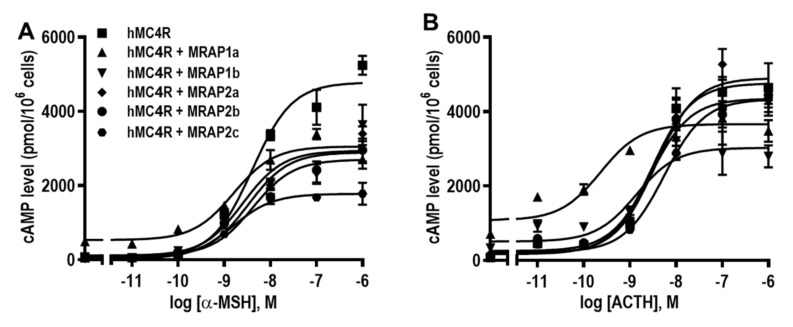
The signaling properties of hMC4R are regulated by hMRAP1s or hMRAP2s in response to α-MSH (**A**) and ACTH (**B**). HEK293T cells were transiently transfected hMC4R with or without MRAP1a, MRAP1b, MRAP2a, MRAP2b or MRAP2c plasmids (1:5), and intracellular cAMP levels were measured by RIA after stimulation with different concentrations of α-MSH or ACTH(1–24). Data are mean ± SEM from triplicate measurements within one experiment. All experiments were performed at least three times independently.

Modulation of hMC4R signaling by MRAP1s and MRAP2s was also studied. Data showed all MRAPs had no effects on α-MSH potencies of hMC4R (Figure 7A and Table 4). Only hMRAP1a significantly increased ACTH potency, and the other MRAPs did not affect ACTH potency at hMC4R (Figure 7B and Table 4). Both hMRAP1a and hMRAP1b significantly increased the basal cAMP levels, whereas all three MRAP2s decreased the basal activities of hMC4R (Table 4). Additionally, all MRAPs decreased R_max_s of hMC4R when α-MSH was used (Figure 7A and Table 4). Only hMRAP1b decreased ACTH-stimulated cAMP generation, and the other MRAPs had no effect on R_max_s of hMC4R in response to ACTH (Figure 7B and Table 4).

## 4. Discussion

Alternative splicing is prevalent in eukaryotes, resulting in a greatly increased diversity of proteins encoded by the genome [59]. Tissue-specific and developmentally regulated alternative splicing is also modulated by divergent stimulation. Approximately 95% of multi-exon genes are alternatively spliced in humans [60,61]. Isoforms produced by alternative splicing might have different functions. For example, two splice variants of receptor expression-enhancing protein 6 gene have distinct functions in the retina [62]. However, in the majority of cases, isoforms from alternative splicing have not been well investigated. In this study, we identified two human *MRAP2* splice variants, *MRAP2b* and *MRAP2c*. Additional studies are needed to confirm which tissues express these alternative splicing variants. Human MRAP1 also has two isoforms: MRAP1a and MRAP1b. The potential effects of the two MRAP1 and three MRAP2 isoforms on hMC3R and hMC4R pharmacology were investigated herein.

Human MRAP1s and hMRAP2s have several similar structural features as MRAP1 and MRAP2 of other species. The conserved motif, LKAHKHS in hMRAP1 or LKAHKYS in hMRAP2, is required for reverse topology (Figure 1B) [31,63,64], and the corresponding reverse topology motif is also observed in MRAP1 and MRAP2 orthologs of other species [65]. In addition, YEYY motif is apparent in both MRAP1 and MRAP2 of nearly every vertebrate examined [66] and plays an important role for MC2R activation [67]. However, the activation motif (LDYL) was only found in the hMRAP1 paralogs but not in hMRAP2s (Figure 1B), which is a critical difference between MRAP1 and MRAP2 [65]. MRAP1 paralogs facilitate the activation of hMC2R, but MRAP2 paralogs (without this activation motif) cannot promote MC2R activation in teleosts and tetrapods [64,65,68,69].

Detailed pharmacological studies were performed on potential MRAP regulation of hMC3R. There was no report on the regulation of hMC3R by hMRAP1b, hMRAP2b, and hMRAP2c. Both hMRAP1a and hMRAP2a were reported to decrease the cell surface expression of hMC3R [35,50]. Our data showed that hMRAP1a and hMRAP2c increased, hMRAP1b decreased, and hMRAP2a and hMRAP2b had no effect on the cell surface expression of hMC3R (Figure 2A). Previously, it has been reported that both hMRAP1a and hMRAP2a decrease NDP-MSH-stimulated [35] or increase α-MSH-induced [37,50] cAMP production of hMC3R. The current study is the first to explore potential MRAP modulation of MC3R using ACTH. In this study, four MRAPs (hMRAP1a, hMRAP1b, hMRAP2a, and hMRAP2c) showed similar effects on MC3R signaling, resulting in decreased α-MSH- and ACTH-stimulated cAMP levels of hMC3R (MRAP2b only decreased ACTH-stimulated signaling of hMC3R) (Figure 4 and Table 2). Our findings indicated that hMRAP1b, hMRAP2b, and hMRAP2c might also be involved in regulating hMC3R in distinct ways compared with hMRAP1a and hMRAP2a. In addition, MRAP1 or MRAP2 has been reported to increase ACTH potency at chicken and frog MC3R [44,70]. However, MRAP2s have no effect on ACTH potency of fish (topmouth culter) MC3R [48]. Our current results showed that all MRAPs had no effect on ACTH potency at hMC3R (Table 2). Further studies in MC3Rs from other species are needed to address whether MRAPs change MC3R to an ACTH-preferring receptor.

The regulation of MRAP1s and MRAP2s on hMC4R was also studied. It was reported that hMRAP1a and hMRAP2a decrease the cell surface expression of hMC4R [35,50]. Our current results showed that hMRAP1a, hMRAP2a, and hMRAP2c increased the cell surface expression of hMC4R whereas hMRAP1b and hMRAP2b had no effect (Figure 5A). For signaling, conflicting results were reported previously: hMRAP1a was reported to either decrease NDP-MSH-stimulated [35] or increases [50,71] or does not affect α-MSH-stimulated [37] signaling of hMC4R. Our data showed that MRAP1b decreased α-MSH- and ACTH-induced cAMP generation, while MRAP1a only decreased α-MSH-stimulated cAMP signaling of hMC4R (Figure 7 and Table 4). Inconsistent results were also reported on hMRAP2-regulated hMC4R signaling: MRAP2a has no effect [51] or increases [50,71] α-MSH-stimulated signaling of hMC4R. MRAP2a does not affect ACTH-induced [51] or decreases NDP-MSH-stimulated [35] cAMP levels of hMC4R. Our study demonstrated that all MRAP2s decreased α-MSH-stimulated cAMP signaling but had no effect on ACTH-induced signaling of hMC4R (Figure 7 and Table 4). Similar results were also observed in chicken MC4R, in which MRAP1 and MRAP2 do not affect ACTH-stimulated signaling but inhibit α-MSH-induced signaling [44]. Our findings suggested that the new isoforms studied herein, hMRAP1b, hMRAP2b, and hMRAP2c, could modulate MC4R signaling.

An interesting observation reported previously is that MRAPs might change MC4R preference to different endogenous ligands. Previous results showed that hMRAP1a or hMRAP2a increase [51,71] or do not affect α-MSH potency at hMC4R [37]. Two endogenous hormones, α-MSH and ACTH, are used in this study to investigate whether MRAPs change ligand potencies of hMC4R. Our results showed that MRAP1s and MRAP2s could not change α-MSH potencies of hMC4R (Table 4). For ACTH, there is no report on whether MRAP1 affects ACTH potency at hMC4R, and hMRAP2a was reported to increase ACTH potency at hMC4R [51]. MRAP2 increase of ACTH potency of MC4R has also been observed in several other species, including pig, chicken, frog, and zebrafish [44,46,51,70,72]. However, this phenomenon was not observed in several other species, such as orange-spotted grouper [73], Nile tilapia [74], topmouth culter [47], and snakehead [49]. Our results showed that only hMRAP1a significantly increased ACTH potency, and the other MRAPs had no effect on ACTH potency at hMC4R (Table 4). We conclude that the MRAP effect on ACTH potency at MC4R might be species-dependent.

Human MC4R has modest basal cAMP signaling [58]. The loss of constitutive activity in MC4R mutations is considered as one cause of obesity [75,76]. The higher constitutive activity of hMC4R is pivotal in regulating energy homeostasis [77] and increased basal activity of MC4R might protect against obesity. Human MRAP1a was shown to increase [37,71] or have no significant effects on the constitutive activity of hMC4R [35,36]. The ratios between hMC4R and hMRAP1a have an important effect on the basal activity of hMC4R [37,71], which might result in the inconsistent results. Our finding showed that both hMRAP1a and hMRAP1b significantly increased hMC4R basal activity (Table 4).

At hMC4R, MRAP2 was reported to have no significant effect on the basal activity [35,36,71,78]. However, MRAP2(s) has been shown to decrease MC4R basal activity in other species, including zebrafish [43], orange-spotted grouper [73], Nile tilapia [74], topmouth culter [47], and snakehead [49]. In addition, hMRAP2a also inhibits the basal activity of ghrelin receptor [79]. Our study found that all three MRAP2s decreased hMC4R basal cAMP signaling (Table 4). MRAP2 regulates GPCR signaling in a dose-dependent manner [43,47,48,70,73,79,80]. Thus, different ratios between hMC4R and hMRAP2 in previous studies might lead to inconsistent results. The potential regulation of constitutive activity in MC4R by MRAPs needs further study.

Splicing variants with different specific domains provide a nature-made opportunity to study the functions of a specific domain. Lab-generated truncated MRAPs indicate that N-terminus, but not C-terminus, of hMRAP1 has crucial roles in regulating hMC2R trafficking and signaling [81], and similar phenomena have been observed in the cells heterologously expressing truncated mouse MRAP1 and hMC2R [31,63]. The present study found that hMRAP1a and hMRAP1b with different C-termini played different roles in regulating hMC3R or hMC4R pharmacology (Table 2 and Table 4), indicating that C-termini of MRAP1 is important for modulation of MC3R and MC4R signaling. Results of MRAP2 deletion mutants and chimeras indicate that the C-terminus of MRAP2a is important for trafficking and signaling of GPCRs, such as ghrelin receptor, orexin receptor, and prokineticin receptor [79,82]. Similar to MRAP1s, hMRAP2a, hMRAP2b, and hMRAP2c are also different in C-termini. However, our study suggested that C-termini of hMRAP2s played distinct roles in regulating MC3R/MC4R trafficking with similar effects on signaling (Figure 2 and Figure 5, Table 2 and Table 4). Collectively, these results suggest that distinct regions of MRAP1s or MRAP2s might have different roles in regulating diverse GPCRs, resulting in increased complexity of MRAPs in modulating GPCRs.

## 5. Conclusions

In summary, MRAP1b and two newly identified *MRAP2* splicing variants, hMRAP2b and hMRAP2c, had potential roles in regulating MC3R and MC4R pharmacology. All MRAPs except MRAP2b decreased α-MSH- and ACTH-stimulated cAMP generation of hMC3R. MRAP1s and MRAP2s showed opposite effects on the basal activity of hMC4R, with MRAP1s increasing and MRAP2s decreasing the basal activities of hMC4R. MRAP1a conferred increased potency for ACTH at the hMC4R whereas the other MRAPs had no effect on ACTH potency. These findings suggest the complexity of MRAPs in modulating MC3R/MC4R and provide a new opportunity for regulating MC3R and MC4R signaling.

## Figures and Tables

**Table 1:** The ligand binding properties of hMC3R regulated by hMRAP1s and hMRAP2s.

	B_max_ (%)	α-MSH Binding	ACTH Binding
		IC_50_ (nM)	IC_50_ (nM)
hMC3R	100	667.50 ± 152.87	85.81 ± 14.16
hMC3R+MRAP1a	85.69 ± 7.43	360.20 ± 47.09	17.49 ± 3.42 ^a^
hMC3R+MRAP1b	63.26 ± 6.19 ^a^	490.84 ± 179.41	42.75 ± 13.05
hMC3R+MRAP2a	91.42 ± 8.31	820.24 ± 177.02	80.64 ± 13.63
hMC3R+MRAP2b	102.49 ± 7.93	895.85 ± 131.95	65.03 ± 10.47
hMC3R+MRAP2c	87.77 ± 6.35	479.51 ± 71.39	80.94 ± 14.78

Values are expressed as the mean ± SEM of at least three independent experiments. ^a^ Significant difference from the parameter of hMC3R, *p* < 0.05.

**Table 2:** The signaling properties of hMC3R regulated by hMRAP1s and hMRAP2s.

		α-MSH		ACTH(1–24)	
	Basal (%)	EC_50_ (nM)	R_max_ (%)	EC_50_ (nM)	R_max_ (%)
hMC3R	100	1.39 ± 0.16	100	4.63 ± 0.81	100
hMC3R+MRAP1a	77.06 ± 5.95 ^b^	1.31 ± 0.28	58.22 ± 9.55 ^a^	1.81 ± 0.65	58.54 ± 8.98 ^b^
hMC3R+MRAP1b	58.25 ± 5.35 ^c^	5.46 ± 1.99	52.41 ± 11.58 ^a^	8.39 ± 2.58	34.26 ± 3.95 ^c^
hMC3R+MRAP2a	74.18 ± 10.50 ^a^	3.82 ± 1.14	39.07 ± 8.66 ^b^	4.91 ± 2.15	46.85 ± 17.28 ^a^
hMC3R+MRAP2b	67.39 ± 7.43 ^b^	1.25 ± 0.36	86.61 ± 10.49	2.07 ± 0.85	66.85 ± 16.92 ^a^
hMC3R+MRAP2c	64.36 ± 11.57 ^a^	1.79 ± 0.32	59.07 ± 6.60 ^b^	3.39 ± 1.19	52.10 ± 16.25 ^a^

Values are expressed as the mean ± SEM of at least three independent experiments. ^a^ Significant difference from the parameter of hMC3R, *p* < 0.05. ^b^ Significant difference from the parameter of hMC3R, *p* < 0.01. ^c^ Significant difference from the parameter of hMC3R, *p* < 0.001.

**Table 3:** The ligand-binding properties of hMC4R regulated by hMRAP1s and hMRAP2s.

		α-MSH	ACTH(1–24)
	B_max_ (%)	IC50 (nM)	IC50 (nM)
hMC4R	100	335.51 ± 32.19	53.62 ± 13.46
hMC4R+MRAP1a	48.96 ± 7.57 ^a^	234.73 ± 79.01	26.16 ± 13.77
hMC4R+MRAP1b	48.01 ± 8.94 ^a^	289.67 ± 77.10	19.08 ± 2.52 ^a^
hMC4R+MRAP2a	143.32 ± 11.76 ^a^	355.93 ± 39.71	123.18 ± 22.12 ^a^
hMC4R+MRAP2b	133.72 ± 14.64	330.42 ± 70.97	55.78 ± 18.24
hMC4R+MRAP2c	132.51 ± 9.31	380.19 ± 87.45	69.64 ± 10.53

Values are expressed as the mean ± SEM of at least three independent experiments. ^a^ Significant difference from the parameter of hMC4R, *p* < 0.05.

**Table 4:** The signaling properties of hMC4R regulated by hMRAP1s and hMRAP2s.

		α-MSH		ACTH(1–24)	
	Basal (%)	EC_50_ (nM)	R_max_ (%)	EC_50_ (nM)	R_max_ (%)
hMC4R	100	5.10 ± 0.80	100	1.81 ± 0.34	
hMC4R+MRAP1a	659.91 ± 97.58 ^c^	4.79 ± 2.31	47.81 ± 6.97 ^a^	0.33 ± 0.05 ^a^	93.28 ± 23.57
hMC4R+MRAP1b	152.41 ± 12.81 ^b^	4.27 ± 1.33	49.20 ± 7.90 ^a^	0.89 ± 0.19	66.29 ± 12.29 ^a^
hMC4R+MRAP2a	55.88 ± 8.92 ^b^	3.10 ± 0.14	56.90 ± 18.75 ^a^	3.20 ± 0.41	102.73 ± 22.28
hMC4R+MRAP2b	66.34 ± 7.52 ^b^	3.26 ± 0.63	45.96 ± 13.34 ^b^	1.89 ± 0.28	121.35 ± 22.98
hMC4R+MRAP2c	65.37 ± 7.55 ^b^	3.43 ± 0.96	33.04 ± 7.10 ^b^	3.27 ± 0.86	81.54 ± 14.07

Values are expressed as the mean ± SEM of at least three independent experiments. ^a^ Significant difference from the parameter of hMC4R, *p* < 0.05. ^b^ Significant difference from the parameter of hMC4R, *p* < 0.01. ^c^ Significant difference from the parameter of hMC4R, *p* < 0.001.

## Data Availability

The raw data supporting the conclusions of this article will be made available by the authors upon request, without undue reservation.
